# Activity of Oral Tebipenem-Avibactam in a Mouse Model of Mycobacterium abscessus Lung Infection

**DOI:** 10.1128/aac.01459-22

**Published:** 2023-01-23

**Authors:** Dereje A. Negatu, Rubén González del Río, Mónica Cacho-Izquierdo, David Barros-Aguirre, Joël Lelievre, Joaquín Rullas, Patricia Casado, Uday S. Ganapathy, Matthew D. Zimmerman, Martin Gengenbacher, Véronique Dartois, Thomas Dick

**Affiliations:** a Center for Discovery and Innovation, Hackensack Meridian Health, Nutley, New Jersey, USA; b Center for Innovative Drug Development and Therapeutic Trials for Africa (CDT-Africa), Addis Ababa University, Addis Ababa, Ethiopia; c Global Health Medicines R&D, GlaxoSmithKline, Tres Cantos, Madrid, Spain; d Department of Medical Sciences, Hackensack Meridian School of Medicine, Nutley, New Jersey, USA; e Department of Microbiology and Immunology, Georgetown University, Washington, DC, USA

**Keywords:** nontuberculous mycobacteria, NTM, beta-lactam, beta-lactamase inhibitor, tebipenem-pivoxil, ARX-1796

## Abstract

The combination of the β-lactam tebipenem and the β-lactamase inhibitor avibactam shows potent bactericidal activity against Mycobacterium abscessus *in vitro*. Here, we report that the combination of the respective oral prodrugs tebipenem-pivoxil and avibactam ARX-1796 showed efficacy in a mouse model of M. abscessus lung infection. The results suggest that tebipenem-avibactam presents an attractive oral drug candidate pair for the treatment of M. abscessus pulmonary disease and could inform the design of clinical trials.

## INTRODUCTION

Mycobacterium abscessus, a member of the nontuberculous mycobacteria (NTM), accounts for most infections caused by fast-growing NTM. When inhaled or acquired by aspiration of contaminated water, M. abscessus can establish extremely difficult-to-cure lung infections (1). NTM infection is a concerning threat to cystic fibrosis (CF) patients, with a prevalence of 5% to 20% (2). M. abscessus is the most common NTM pathogen in CF patients (3), and disease is associated with rapidly worsening lung function (4). M. abscessus lung disease is difficult to treat, requiring complex antibiotic regimens, administered often for years, with low cure rates. The regimens include injectables and have serious side effects, including ototoxicity and impaired liver function (5). Several antibiotics cause pharmacological drug-drug interactions, further limiting therapeutic options. Lung transplantation is often contraindicated in the presence of M. abscessus, and mortality is high (6, 7). Desperate clinicians and patients are turning to experimental therapy with mycobacteriophages due to the lack of effective drugs (8). In brief, against M. abscessus lung disease, there is no reliable cure (9), and new oral, well-tolerated, bactericidal drugs are sorely needed (10).

**FIG 1:**
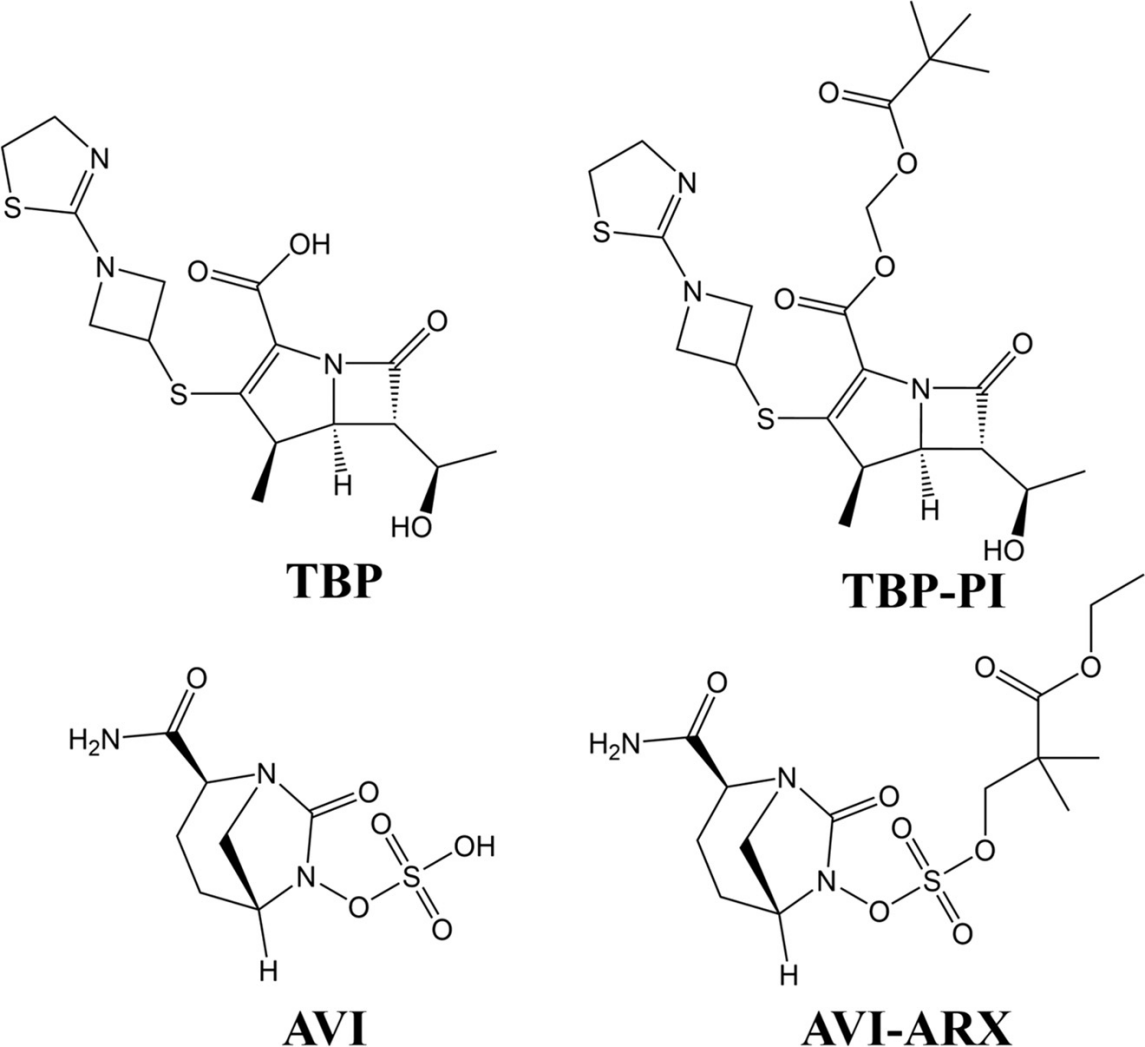
Structure of tebipenem and avibactam and their prodrug forms. TBP, bioactive form of tebipenem; AVI, bioactive form of avibactam; TBP-PI, tebipenem-pivoxil (prodrug form of tebipenem); AVI-ARX, ARX-1796 (prodrug form of avibactam) (21, 22).

β-Lactams are bactericidal and generally well tolerated (11). Two β-lactams, imipenem (IPM) and cefoxitin (FOX), used to treat M. abscessus lung disease, are administered by the intravenous route and show modest *in vitro* activity at 20 to 50 μM, limiting their clinical utility (12). In contrast to IPM and FOX, the β-lactam tebipenem (TBP) in combination with the β-lactamase inhibitor avibactam (AVI) exhibits an attractive, low micromolar MIC and pronounced bactericidal activity *in vitro* (13–16). However, both TBP and AVI show limited oral bioavailability (17, 18), reducing their clinical attractiveness. An orally bioavailable prodrug of tebipenem, tebipenem-pivoxil (TBP-PI) (Fig. 1), has been approved for the treatment of pediatric respiratory diseases in Japan (19) and has entered phase III clinical trials in the United States for the treatment of urinary tract infections (20) (ClinicalTrials.gov identifier NCT03788967). An oral prodrug of avibactam, ARX-1796 (also known as AV-006; abbreviated here AVI-ARX) (Fig. 1), is currently in a phase I clinical trial (21) (ClinicalTrials.gov identifier NCT03931876). Thus, the combination of TBP-PI plus AVI-ARX presents a potential repurposing opportunity for an oral bactericidal drug duo for the treatment of M. abscessus lung disease.

**FIG 2:**
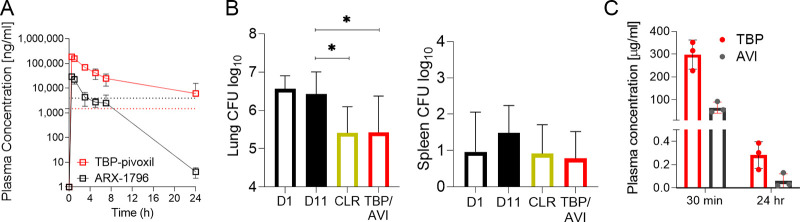
Pharmacokinetic profile and efficacy of the tebipenem prodrug TBP-PI combined with the avibactam prodrug AVI-ARX in mice. (A) Plasma concentration-time profile of TBP, the active component of TBP-PI, and AVI, the active component of AVI-ARX, following a single oral dose of 400 mg/kg TBP-PI (EC/13222; GlaxoSmithKline, Tres Cantos, Madrid, Spain) and 200 mg/kg AVI-AXR (catalog number HY-132987; MedChemExpress) in CD1 mice. The red dotted line shows the MIC of TBP (4 μM; 1.5 μg/mL) in the presence of 4 μg/mL AVI (indicated by the black dotted line) against M. abscessus K21 (16). (B) Efficacy of TBP-PI plus AVI-ARX (TPB/AVI) and clarithromycin (CLR) control in an NOD SCID mouse model. Mouse lung (left) and spleen (right) CFU are shown 1 day after intranasal infection with M. abscessus K21 (D1), following daily oral vehicle 0.5% carboxymethyl cellulose with 0.2% Tween 80 (TBP-PI plus AVI-AXR vehicle) for 10 days (D11), 6 times twice-daily oral administration followed by 4 days of once-daily oral administration of 400 mg/kg TBP-PI plus 200 mg/kg AVI-AXR (TBP/AVI), or daily oral administration of clarithromycin (250 mg/kg formulated in 0.5% carboxymethyl cellulose) for 10 days. The mean and standard deviation are shown for each treatment group (*n* = 6). Statistical significance of the results was analyzed by one-way analysis of variance (ANOVA) multicomparison and Dunnett’s posttest; *, *P* < 0.05. (C) Plasma concentration monitoring of TBP and AVI in infected NOD SCID mice 30 min and 24 h after the last dose in the efficacy experiment shown in panel B.

To determine whether the attractive *in vitro* activities of TBP plus AVI translate into *in vivo* efficacy when administered as an oral combination of TBP-PI plus AVI-ARX, an immunodeficient murine model based on NOD.CB17-*Prkdc^scid^*/NCrCrl mice (NOD SCID; Charles River Laboratories) was utilized (23). In this model, mice were infected with the recent M. abscessus clinical isolate K21 to generate a sustained infection, resulting in a largely constant bacterial lung burden, thus allowing the effects of drugs to be evaluated (23). M. abscessus K21 belongs to the subspecies *abscessus*, forms rough colonies, and harbors the C28 sequevar of *erm41*; it is thus macrolide sensitive (23). The MIC of TBP (in the presence of 4 μg/mL AVI) against isolate K21 was 4 μM (16). To determine the appropriate dosing, the plasma concentration-time profile of TBP-PI and AVI-ARX administered orally to CD1 mice (Charles River Laboratories) was determined by liquid chromatography coupled with tandem mass spectrometry. Pharmacokinetic-pharmacodynamic data analysis revealed that dosing with 400 mg/kg TBP-PI provided concentrations over the TBP MIC (in the presence of 4 μg/mL AVI) for the entire 24-h dosing interval, and 200 mg/kg AVI-ARX yielded concentrations of over 4 μg/mL for 4 to 7 h following a single dose (Fig. 2A). Eight-week-old female NOD SCID mice were infected intranasally with 10^6^ CFU of M. abscessus K21 as described previously (23). TBP-PI and AVI-ARX were administered orally first, twice daily for 6 days at 400 and 200 mg/kg, respectively, starting 1 day postinfection. As reduced appetite was observed, the dosing frequency was reduced to once daily from days 7 to 10, which reverted this effect. Clarithromycin was administered orally once daily as a positive control at 250 mg/kg, as described previously (24). We typically employ clarithromycin as a positive control in our drug discovery projects (16, 23), as this is the macrolide recommended for macrolide drug susceptibility testing (25). However, the macrolide azithromycin is preferred clinically due to its improved tolerability, fewer drug-drug interactions, and equal efficacy (26, 27). All experiments involving live animals were approved by the Institutional Animal Care and Use Committee of the Center for Discovery and Innovation, Hackensack Meridian Health, and were conducted in accordance with the GSK Policy on the Care, Welfare and Treatment of Laboratory Animals and reviewed by the Institutional Animal Care and Use Committee either at GSK or by the ethical review process at the institution where the work was performed.

All mice were euthanized 24 h after the last dose. Lung and spleen bacterial loads were quantified by plating serial dilutions of organ homogenates onto Middlebrook 7H11 agar. Treatment with the vehicle alone did not affect the bacterial lung burden (“D11 vehicle”; Fig. 2B). Compared to the vehicle control, treatment with TBP-PI plus AVI-ARX reduced the lung CFU by ~10-fold (Fig. 2B). CFU reduction in the spleen followed a similar pattern but was not statistically significant (Fig. 2B). Drug levels were measured 30 min and 24 h after the last dose, confirming on-target concentrations in the infected NOD SCID mice (Fig. 2A and C). Thus, the combination of the prodrug forms of TBP and AVI is efficacious in a mouse model of M. abscessus lung infection.

Treatment of M. abscessus lung disease requires multidrug regimens (9). To detect potential antagonism between the TBP-plus-AVI combination and approved drugs or clinical development candidates, we carried out *in vitro* checkerboard analyses with the type strain M. abscessus ATCC 19977 as previously described (28), combining TBP (plus AVI) with clarithromycin, amikacin, imipenem, cefoxitin, tigecycline, tedizolid, omadacycline, clofazimine, bedaquiline, moxifloxacin, SPR719, epetraborole, and rifabutin (Table 1). The combination of imipenem (IPM) with TBP plus AVI was synergistic. The remaining combinations were additive (Table 1). The absence of antagonistic interactions suggests that TBP-PI plus AVI-ARX can be coadministered with either of these drugs without affecting each other’s activity.

**TABLE 1:** Drug-drug potency interactions between TBP/AVI and anti-M. abscessus approved drugs or clinical candidates

Drug(s)^*a*^	Class	Target	MIC (μM)		FICI^*b*^
			Alone	Combined	
TBP/AVI	Carbapenem	Peptidoglycan biosynthesis	3.0	0.8	0.77
Clarithromycin	Macrolide	50S ribosomal subunit	3.0	1.5	
TBP/AVI			3.0	1.0	0.83
Amikacin	Aminoglycoside	30S ribosomal subunit	12.5	6.3	
TBP/AVI			3.0	0.2	0.47
Imipenem	Carbapenem	Peptidoglycan biosynthesis	15.0	6.0	
TBP/AVI			3.0	1.0	0.83
Cefoxitin	Cephalosporin	Peptidoglycan biosynthesis	25.0	12.5	
TBP/AVI			3.0	1.5	0.88
Tigecycline	Glycylcycline	30S ribosomal subunit	16.0	6.0	
TBP/AVI			3.0	0.8	0.54
Tedizolid	Oxazolidinone	50S ribosomal subunit	3.0	0.8	
TBP/AVI			3.0	1.5	0.74
Omadacycline	Tetracycline	30S ribosomal subunit	12.5	3.0	
TBP/AVI			3.0	1.0	0.97
Clofazimine	Riminophenazine	Electron transport chain	12.5	8.0	
TBP/AVI			3.0	1.2	0.65
Bedaquiline	Diarylquinoline	ATP synthase	0.8	0.2	
TBP/AVI			3.0	0.8	0.77
Moxifloxacin	Fluoroquinolone	DNA gyrase	3.0	1.5	
TBP/AVI			3.0	1.5	0.77
SPR719	Benzimidazole	DNA gyrase ATPase subunit	1.5	0.4	
TBP/AVI			3.0	1.5	0.90
Epetraborole	Benzoxaborole	Leucyl-tRNA synthetase	0.5	0.2	
TBP/AVI			3.0	0.5	0.59
Rifabutin	Rifamycin	RNA polymerase	1.2	0.5	

a To determine potency interactions between TBP/AVI and other drugs, checkerboard analyses were carried out as previously described (28), using M. abscessus ATCC 19977 in a 96-well plate format and the optical density at 500 nm (OD_500_) as the readout for growth. The effect of serially diluted TBP at doses ranging from 12.5 to 0.012 μM plus a 4-μg/mL fixed concentration of AVI was tested against the partner drugs at doses ranging from 25 to 0.2 μM. The experiment was repeated once, yielding the same results. TBP, tebipenem; AVI, avibactam. TBP, catalog number 161715-21-5 (MuseChem); AVI, catalog number HY-14879A (MedChemExpress); clarithromycin, catalog number C9742 (Sigma-Aldrich); amikacin, catalog number PHR1654 (Sigma-Aldrich); imipenem, catalog number PHR 1796 (Sigma-Aldrich); cefoxitin, catalog number C4786 (Sigma-Aldrich); tigecycline, catalog number HY-B0117 (MedChemExpress); tedizolid, catalog number HY-14855 (MedChemExpress); omadacycline, catalog number HY-14865 (MedChemExpress); clofazimine, catalog number HY-B1046 (MedChemExpress); bedaquiline, catalog number HY-14881 (MedChemExpress); moxifloxacin, catalog number SML1581 (Sigma-Aldrich); SPR719, catalog number HY-12930 (MedChemExpress); epetraborole, catalog number HY-12479A (MedChemExpress); rifabutin, catalog number HY-17025 (MedChemExpress).

b The fractional inhibitory concentration index (FICI) was calculated by using the concentrations at which at least 90% growth inhibition of the cultures compared to the drug-free culture was observed. FICI = [(concentration of drug A in combination/concentration of drug A alone) + (concentration of drug B in combination/concentration of drug B alone)]. FICI values are as follows: ≤0.5, synergistic; 0.5 to 1.0, additive; >1.0 to <2.0, indifferent; ≥2, antagonistic (30).

One limitation of this study is the small fraction of the dosing interval during which the concentration of AVI-ARX was higher than 4 μg/mL following a single daily dose, i.e., from days 7 to 10 in the efficacy study. This concentration is clinically relevant and protects TBP against the major β-lactamase activity in M. abscessus (16, 29). Therefore, we may have slightly underestimated the efficacy of tebipenem. Our *in vitro* data indicate that the MIC of TBP was 4 μM (1.5 μg/mL) in the presence of 4 μg/mL or 2 μg/mL AVI, and the 2 μg/mL threshold was achieved in plasma for 8 h postdose (Fig. 2A).

In conclusion, the oral form of TBP, coadministered with the oral form of AVI, is active against M. abscessus in a mouse model of lung infection. Thus, this work adds an oral bactericidal drug candidate pair to the M. abscessus pipeline. *In vitro* potency interaction studies suggest that TBP plus AVI synergizes with the standard of care β-lactam IPM and does not antagonize the activity of the remaining antibiotics in clinical use or in development against M. abscessus lung disease. We note that TBP plus AVI in combination with orally bioavailable amoxicillin was recently shown to exert remarkable bactericidal synergy *in vitro* (16). Clinical trials are warranted to (i) determine the utility of oral TBP plus AVI and (ii) test the dual β-lactam combination of TBP (plus AVI) and amoxicillin in patients suffering from M. abscessus lung disease. Given the expense and risks of intravenous therapies that necessitate shorter courses than needed for eradication, these combinations as oral drug options would provide huge advantages (31).

## References

[1] Johansen MD, Herrmann JL, Kremer L. 2020. Non-tuberculous mycobacteria and the rise of Mycobacterium abscessus. Nat Rev Microbiol 18:392–407. 10.1038/s41579-020-0331-1. 32086501

[2] Qvist T, Pressler T, Høiby N, Katzenstein TL. 2014. Shifting paradigms of nontuberculous mycobacteria in cystic fibrosis. Respir Res 15:41. 10.1186/1465-9921-15-41. 24725650 PMC3986433

[3] Abidin NZ, Gardner AI, Robinson HL, Haq IJ, Thomas MF, Brodlie M. 2021. Trends in nontuberculous mycobacteria infection in children and young people with cystic fibrosis. J Cyst Fibros 20:737–741. 10.1016/j.jcf.2020.09.007. 32950411 PMC8490157

[4] Qvist T, Taylor-Robinson D, Waldmann E, Olesen HV, Hansen CR, Mathiesen IH, Høiby N, Katzenstein TL, Smyth RL, Diggle PJ, Pressler T. 2016. Comparing the harmful effects of nontuberculous mycobacteria and Gram negative bacteria on lung function in patients with cystic fibrosis. J Cyst Fibros 15:380–385. 10.1016/j.jcf.2015.09.007. 26482717 PMC4893021

[5] Hughes DA, Bokobza I, Carr SB. 2021. Eradication success for non-tuberculous mycobacteria in children with cystic fibrosis. Eur Respir J 57:2003636. 10.1183/13993003.03636-2020. 33542059 PMC8280568

[6] Kavaliunaite E, Harris KA, Aurora P, Dixon G, Shingadia D, Muthialu N, Spencer H. 2020. Outcome according to subspecies following lung transplantation in cystic fibrosis pediatric patients infected with Mycobacterium abscessus. Transpl Infect Dis 22:e13274. 10.1111/tid.13274. 32129923

[7] Brugha R, Spencer H. 2021. Mycobacterium abscessus in cystic fibrosis. Science 372:465–466. 10.1126/science.abi5695. 33926941

[8] Dedrick RM, Guerrero-Bustamante CA, Garlena RA, Russell DA, Ford K, Harris K, Gilmour KC, Soothill J, Jacobs-Sera D, Schooley RT, Hatfull GF, Spencer H. 2019. Engineered bacteriophages for treatment of a patient with a disseminated drug-resistant Mycobacterium abscessus. Nat Med 25:730–733. 10.1038/s41591-019-0437-z. 31068712 PMC6557439

[9] Daley CL, Iaccarino JM, Lange C, Cambau E, Wallace RJ, Jr, Andrejak C, Böttger EC, Brozek J, Griffith DE, Guglielmetti L, Huitt GA, Knight SL, Leitman P, Marras TK, Olivier KN, Santin M, Stout JE, Tortoli E, van Ingen J, Wagner D, Winthrop KL. 2020. Treatment of nontuberculous mycobacterial pulmonary disease: an official ATS/ERS/ESCMID/IDSA clinical practice guideline. Eur Respir J 56:2000535. 10.1183/13993003.00535-2020. 32636299 PMC8375621

[10] Egorova A, Jackson M, Gavrilyuk V, Makarov V. 2021. Pipeline of anti-Mycobacterium abscessus small molecules: repurposable drugs and promising novel chemical entities. Med Res Rev 41:2350–2387. 10.1002/med.21798. 33645845 PMC8217127

[11] Story-Roller E, Maggioncalda EC, Cohen KA, Lamichhane G. 2018. Mycobacterium abscessus and β-lactams: emerging insights and potential opportunities. Front Microbiol 9:2273. 10.3389/fmicb.2018.02273. 30319581 PMC6167491

[12] Lavollay M, Dubée V, Heym B, Herrmann JL, Gaillard JL, Gutmann L, Arthur M, Mainardi JL. 2014. In vitro activity of cefoxitin and imipenem against Mycobacterium abscessus complex. Clin Microbiol Infect 20:O297–O300. 10.1111/1469-0691.12405. 24112243

[13] Kaushik A, Gupta C, Fisher S, Story-Roller E, Galanis C, Parrish N, Lamichhane G. 2017. Combinations of avibactam and carbapenems exhibit enhanced potencies against drug-resistant Mycobacterium abscessus. Future Microbiol 12:473–480. 10.2217/fmb-2016-0234. 28326811 PMC5618940

[14] Gumbo T, Cirrincione K, Srivastava S. 2020. Repurposing drugs for treatment of Mycobacterium abscessus: a view to a kill. J Antimicrob Chemother 75:1212–1217. 10.1093/jac/dkz523. 32016429

[15] Kumar P, Chauhan V, Silva JRA, Lameira J, d'Andrea FB, Li S-G, Ginell SL, Freundlich JS, Alves CN, Bailey S, Cohen KA, Lamichhane G. 2017. Mycobacterium abscessus l,d-transpeptidases are susceptible to inactivation by carbapenems and cephalosporins but not penicillins. Antimicrob Agents Chemother 61:e00866-17. 10.1128/aac.00866-17. 28760902 PMC5610527

[16] Negatu DA, Zimmerman MD, Dartois V, Dick T. 2022. Strongly bactericidal all-oral β-lactam combinations for the treatment of Mycobacterium abscessus lung disease. Antimicrob Agents Chemother 66:e0079022. 10.1128/aac.00790-22. 36047786 PMC9487536

[17] Fernández ÁE, Voong VP, de Cozar C, Willé DR, Urones B, Cortés A, Price A, Tran D, Hoang N, Ha TT, McCloskey M, Shaheen S, Dayao D, de Mercado J, Castañeda P, García-Perez A, Singa B, Pavlinac P, Walson J, Martínez-Martínez MS, Arnold SLM, Saul T, Ballell PL, Baker S. 2022. The repurposing of tebipenem pivoxil as alternative therapy for severe gastrointestinal infections caused by extensively drug-resistant Shigella spp. Elife 11:e69798. 10.7554/eLife.69798. 35289746 PMC8959600

[18] Crass RL, Pai MP. 2019. Pharmacokinetics and pharmacodynamics of β-lactamase inhibitors. Pharmacotherapy 39:182–195. 10.1002/phar.2210. 30589457

[19] Sodhi V, Kronsberg KA, Clark M, Cho JC. 2021. Tebipenem pivoxil hydrobromide—no PICC, no problem! Pharmacotherapy 41:748–761. 10.1002/phar.2614. 34370326

[20] Eckburg PB, Muir L, Critchley IA, Walpole S, Kwak H, Phelan AM, Moore G, Jain A, Keutzer T, Dane A, Melnick D, Talley AK. 2022. Oral tebipenem pivoxil hydrobromide in complicated urinary tract infection. N Engl J Med 386:1327–1338. 10.1056/NEJMoa2105462. 35388666

[21] Gordon EM, Duncton MAJ, Gallop MA. 2018. Orally absorbed derivatives of the β-lactamase inhibitor avibactam. Design of novel prodrugs of sulfate containing drugs. J Med Chem 61:10340–10344. 10.1021/acs.jmedchem.8b01389. 30296086

[22] Isoda T, Ushirogochi H, Satoh K, Takasaki T, Yamamura I, Sato C, Mihira A, Abe T, Tamai S, Yamamoto S, Kumagai T, Nagao Y. 2006. Syntheses and pharmacokinetic studies of prodrug esters for the development of oral carbapenem, L-084. J Antibiot (Tokyo) 59:241–247. 10.1038/ja.2006.34. 16830892

[23] Dick T, Shin SJ, Koh W-J, Dartois V, Gengenbacher M. 2020. Rifabutin is active against Mycobacterium abscessus in mice. Antimicrob Agents Chemother 64:e01943-19. 10.1128/aac.01943-19. 31767722 PMC6985736

[24] Aragaw WW, Roubert C, Fontaine E, Lagrange S, Zimmerman MD, Dartois V, Gengenbacher M, Dick T. 2022. Cyclohexyl-griselimycin is active against Mycobacterium abscessus in mice. Antimicrob Agents Chemother 66:e0140021. 10.1128/AAC.01400-21. 34723632 PMC8765428

[25] Clinical and Laboratory Standards Institute. 2018. Susceptibility testing of mycobacteria, Nocardia spp., and other aerobic actinomycetes, 3rd ed. CLSI document M24. Clinical and Laboratory Standards Institute, Wayne, PA. 31339680

[26] Pathak K, Hart S, Lande L. 2022. Nontuberculous mycobacteria lung disease (NTM-LD): current recommendations on diagnosis, treatment, and patient management. Int J Gen Med 15:7619–7629. 10.2147/IJGM.S272690. 36213301 PMC9534142

[27] Griffith DE, Daley CL. 2022. Treatment of Mycobacterium abscessus pulmonary disease. Chest 161:64–75. 10.1016/j.chest.2021.07.035. 34314673

[28] Aziz DB, Teo JWP, Dartois V, Dick T. 2018. Teicoplanin-tigecycline combination shows synergy against Mycobacterium abscessus. Front Microbiol 9:932. 10.3389/fmicb.2018.00932. 29867841 PMC5958212

[29] Dubée V, Bernut A, Cortes M, Lesne T, Dorchene D, Lefebvre A-L, Hugonnet J-E, Gutmann L, Mainardi J-L, Herrmann J-L, Gaillard J-L, Kremer L, Arthur M. 2015. β-Lactamase inhibition by avibactam in Mycobacterium abscessus. J Antimicrob Chemother 70:1051–1058. 10.1093/jac/dku510. 25525201

[30] European Committee for Antimicrobial Susceptibility Testing of the European Society of Clinical Microbiology and Infectious Diseases. 2000. EUCAST definitive document E.Def 1.2, May 2000: terminology relating to methods for the determination of susceptibility of bacteria to antimicrobial agents. Clin Microbiol Infect 6:503–508. 10.1046/j.1469-0691.2000.00149.x. 11168186

[31] Dartois V, Dick T. 2022. Drug development challenges in nontuberculous mycobacterial lung disease: TB to the rescue. J Exp Med 219:e20220445. 10.1084/jem.20220445. 35543723 PMC9098649

